# The latest advancements in Sosuga virus (SOSV) research

**DOI:** 10.3389/fmicb.2024.1486792

**Published:** 2024-11-01

**Authors:** Nathan M. Markarian, Levon Abrahamyan

**Affiliations:** ^1^Faculté de Pharmacie, Université de Montréal, Montréal, QC, Canada; ^2^Laboratory of Animal Molecular Virology, Swine and Poultry Infectious Diseases Research Center (CRIPA) and Research Group on Infectious Diseases in Production Animals (GREMIP), Faculté de Médecine Vétérinaire, Université de Montréal, Saint-Hyacinthe, QC, Canada

**Keywords:** Sosuga virus, paramyxovirus, viral spillover, *Rousettus aegyptiacus*, Egyptian rousette bat, viral zoonoses, emerging viral disease

## Abstract

The last 60 years have seen the emergence of several zoonotic viruses, some of which originate from bats. Among these are Nipah virus, Marburg virus and Ebola viruses, which have high case fatality rates, and pose significant public health risks. In 2012, another zoonotic paramyxovirus from bats, known as Sosuga Virus (SOSV), was discovered in a hospitalized biologist who had returned from a trip to Africa. Given the potential public health threats of the SOSV, investigating its pathogenesis, epidemiology and developing antiviral strategies are crucial to control possible future outbreaks. Thus, in this review, we explore the latest advancements in understanding SOSV since its discovery, focusing on its pathogenesis, animal models and the development of antiviral strategies. By examining the current literature, this review aims to provide a comprehensive overview to guide future studies and help public health efforts in better mitigating potential SOSV outbreaks.

## 1 Introduction

Zoonotic diseases cause up to 61% of infectious in humans (Rahman et al., 2020; Taylor et al., 2001) and 71.8% of these zoonotic diseases originate in wildlife (Jones et al., 2008). As witnessed by several outbreaks, including the global Coronavirus disease-19 (COVID-19) pandemic, zoonotic diseases pose a significant threat to public health. One of the important zoonotic host-reservoir for viruses are bats, which make up 25% of mammalian species and harbor a high viral diversity compared to the other mammalian orders (Han et al., 2015; O’Shea et al., 2014). In fact, many viruses that have emerged in the last 60 years, including Marburg, Hendra and Nipah viruses have bats as their natural reservoir hosts, and are part of the World Health Organization Blueprint List of Priority Diseases (Friedrich, 2018; Letko et al., 2020). The latter viruses have been reported to have high case fatality rates in humans, between 40 and 75% for Nipah virus, around 57% for Hendra virus and 24–88% for Marburg virus (Epstein et al., 2006; Halpin and Rota, 2015; Srivastava et al., 2023). Zoonotic outbreaks caused by bats in the last decades have been primarily reported on the Asian and African continents, which could be due to varying factors namely: an expanding human population, deforestation, biodiverse environments with a large pool of pathogens, migration of rural population into the urban areas, human behaviors, i.e., bushmeat activities in sub-Saharan Africa and consumption of raw palm date sap in rural areas of Bangladesh (Khan et al., 2022). For this reason, several surveillance programs have been set up to probe zoonotic spillovers to humans, although a more proactive approach of probing animal infections prior to human transmissions have been suggested (Sharan et al., 2023).

In the last decade, a new virus, named Sosuga virus (SOSV), of the same family as Nipah virus, Hendra virus and Mumps virus was discovered from Africa, in a 25-year-old female wildlife biologist after she returned from a 6-week field expedition from South Sudan and Uganda (Albariño et al., 2014). During her trip, she had been in contact with more than 20 species of bats and rodents while taking necessary precautions and preventions including vaccines and malarial prophylaxis (Albariño et al., 2014). After being tested negative for a variety of suspected viruses, deep-sequencing and metagenomic analysis of blood samples revealed a negative single stranded RNA virus part of the *Paramyxoviridae* family of around 15 kilobases long and was named Sosuga virus (SOSV) for its probable origin (South Sudan Uganda) (Amman et al., 2015). As paramyxovirus of the genus subfamily *Rubulavirinae* and genus *Pararubulavirus*, SOSV contains for 6 genes (*n, v/p, m, f, hn*, and *l*) which encode for 7 viral proteins: nucleocapsid (N), V protein (V), phosphoprotein (P), matrix protein (M), fusion protein (F), hemagglutinin–neuraminidase (HN), and polymerase (L), forming pleomorphic virions (Albariño et al., 2014; Rima et al., 2019). Both HN and F proteins are found on the surface of paramyxoviruses, the HN protein allows host cell receptor recognition and the F protein the subsequent fusion with the host cell (Jardetzky and Lamb, 2014). Unlike other paramyxoviruses, SOSV does not have a conserved amino acid “NRKSCS” motif in its HN receptor binding protein (RBP) known to bind and hydrolyzing sialic acid presented on host cells. This is why it has been suggested that SOSV utilizes a novel and unknown host entry cell pathway (Stelfox and Bowden, 2019).

## 2 Potential SOSV reservoir

Since the incubation periods of paramyxoviruses range between 1 and 3 weeks (Playford et al., 2010) and that the first SOSV positive patient was in Kibaale, Uganda 3 weeks prior to her symptoms, this prompted an investigation of the origin of this virus at that site (Amman et al., 2015). After capturing and necropsying hundreds of bats in 3 collection sites over a 3 year span, Egyptian rousette bats (ERB) or *Rousettus aegyptiacus*, pooled bat liver and spleen samples were shown to be positive for SOSV and sequences identical to the one from the patient were also detected (Amman et al., 2015). This allowed the researchers to conclude that SOSV was likely originating from ERBs (Amman et al., 2015). Recently, SOSV was detected in the serum and pooled liver, spleen, axillary lymph node, and salivary gland samples of ERBs in the Moyamba District of Sierra Leone, which raises public health concerns as there is a significant range extension of the virus from East to West Africa, throughout the sub-Saharan range (Amman et al., 2024). It is also important to keep in mind that the existing studies may have introduced sample bias, leading to an overrepresentation of bat infections in the specific locations, during particular time-frames and due to the collection strategy used for pooled bat excreta samples (Cohen et al., 2023; Flaquer et al., 2007; Giles et al., 2021). In order to solidify the evidence that ERBs are the reservoir of SOSV, more testing is required throughout the region (Amman et al., 2024).

## 3 SOSV diagnostic strategies

Two main strategies have been developed to detect SOSV natural or experimental infection in bats (Amman et al., 2015; Amman et al., 2020; Amman et al., 2024), as well as experimental infection of hamsters (Welch et al., 2023). One of these strategies is by using a TaqMan real-time RT-PCR selective for the N gene, which was developed after the discovery of SOSV (Albariño et al., 2014). The other method consists of doing an indirect ELISA for the presence of SOSV reactive IgG antibodies using a non-recombinant, infectious-based virus antigen lysate (Amman et al., 2020). Despite a reduced sensitivity, in the advent of SOSV outbreaks, a field-based rapid diagnostic assay, such as lateral flow immunoassays (LFIs) would be of great use to triage SOSV infected patients on the field, especially in areas where electrical power is limited, and with the shortage of trained personnel able to conduct molecular-based assays (Phan et al., 2016).

## 4 SOSV pathogenesis

Thus far, the only patient that has been reported to be infected with SOSV was admitted with symptoms of malaise, headache, generalized myalgia and arthralgia, neck stiffness, a sore throat and a fever which lasted until hospital day 9, and was discharged on day 14 (Albariño et al., 2014). In order to understand how SOSV causes disease, animal models could provide insight, especially in the context of developing antivirals and vaccines. When 2-day old suckling mice were intracranially (IC) and intraperitoneally (IP) infected with blood samples obtained from the patient, 2 out of 20 mice developed neurological symptoms at 9–10 days post infection (DPI), with viral RNA detected in the brain, confirming infection (Albariño et al., 2014). In another study, Syrian hamsters aged 5 weeks were infected intranasally (IN) or IP with recombinant SOSV (rSOSV) or SOSV expressing the ZsGreen1 (ZsG) reporter gene (rSOSV/ZsG) (Welch et al., 2023). Interestingly, all infected animals survived, with no clinical signs of infection, despite detecting viral RNA in several tissues, blood and mucosal swabs. Increased immunoglobulin G (IgG) antibody responses were noted between 8 and 28 days post infection and in IN infected hamsters, lung pathology was observed and viral RNA was detected *in situ* (Welch et al., 2023). In another study, similar results were found in ERBs infected subcutaneously with rSOSV, with no overt clinical illness and presence of virus in a variety of tissues. Mild lesions in the gastrointestinal tract, as well as salivary glands were found and viral loads were shown to increase with time in urine, oral and rectal swabs, which were not observed in the Syrian hamster study (Amman et al., 2020). Histology findings revealed mild to moderate lesions and presence of nucleoprotein (NP) antigen persisting over time in different tissues, with mononuclear phagocyte and CD3+ cell responses in salivary glands at 21 DPI, suggesting a chronic infection (Kirejczyk et al., 2022). To understand the immune mechanism of protection in bats, Schuh et al. (2019) inoculated ERBs with rSOSV/ZsG and recuperated their sera 3 weeks post infection. Amongst the infected bats (*n* = 3), convalescent plasma was only able to neutralize SOSV in presence of guinea pig complement (2/3 bats), leading the authors to suggest that the immune mechanism of protection does not seem to be due to virus-specific neutralizing antibodies (Schuh et al., 2019). In order to warrant such conclusion however, a stronger number of convalescent sera should be analyzed, post-secondary or post-tertiary exposure to SOSV. Going forward in SOSV animal studies, infections in immunodeficient mice such as type I interferon receptor knockout (IFNAR-KO) and other animal species, i.e., guinea pigs, ferrets and nonhuman primates used to study NiV infections could be a potential avenue in investigating SOSV pathogenesis (Pigeaud et al., 2023).

## 5 Discussion: antiviral strategies and future avenues

To prevent a scenario experienced by the wildlife biologist, therapeutics against SOSV are of crucial importance. In Welch et al. (2018) developed a SOSV minigenome segment with the minimum requirements to initiate paramyxovirus replication and transcription and tested a panel of 40 compounds to inhibit these processes. They identified three compounds, two of which are nucleoside analogs: 6-azauridine (EC50 = 7.99 ± 1.32 μM), 2′-deoxy-2′-fluorocytidine (EC50 = 1.48 ± 0.22 μM) and one being an inosine monophosphate dehydrogenase (IMPDH) inhibitor - mycophenolic acid, MPA (0.33 ± 0.04 μM) to inhibit the replication of rSOSV and rSOSV/ZsG in the Huh7 cell line (Welch et al., 2018). Ribavarin (6.10 ± 0.09 μM), another nucleoside inhibitor better studied in NiV was also able to inhibit rSOSV/ZsG replication, along with 09167 (0.67 ± 0.05 μM), an innate immune antagonist previously used to test other paramyxoviruses (Welch et al., 2018). In another study, nucleoside analogs, including remdesivir/RDV/Veklury/GS-5734 (0.052 ± 0.01 μM in Huh7), RDV parent nucleoside RVn/GS-441524 (2.06 ± 0.09 μM in Huh7) and an orally available form of RDV, ODBG-P-RVn (0.52 ± 0.10 μM in Huh7) showed promising antiviral activity against SOSV *in vitro* (Lo Michael et al., 2021). Besides small molecules, Parrington et al. (2023) obtained leukapheresis samples from the only infected patient, 5 years after infection, allowing them to isolate peripheral blood monocyte cells (PBCMs) from which they were able to produce hybridoma cell lines secreting monoclonal antibodies (mAbs). After expressing HN and F SOSV protein antigens in Vero cells, they were able to isolate 24 SOSV-reactive mAbs which were then tested against soluble forms of both HN and F proteins that they engineered, *in vitro*. It was shown that from the 6 anti-HN mAbs isolated, each of them recognized at least 4 distinct antigenic sites of the head domain, neutralizing rSOSV/ZsG infection in Vero cells at an IC50 between 0.4 and 55 ng/μL (Parrington et al., 2023). As for the anti-F mAbs identified, prefusion-specific mAbs (IC50 between 21 and 480 ng/μL) demonstrated greater neutralization than postfusion-specific mAbs (IC50 > 10,000 ng/μL) and many of the pre- and postfusion-specific mAbs (IC50 between 82 and 558 ng/μL), which could be due to the fact that paramyxovirus virions have the prefusion form of the F protein, whereas the postfusion-specific form occurs after fusing with the host cell membrane (Parrington et al., 2023). From these results, the authors suggested that the HN mAb could be used as a therapeutic agent due to its low IC50 of 0.4 ng/mL, and that the mAbs described in their study could be used for further research in the viral receptor of SOSV, as well as vaccine development (Parrington et al., 2023). Indeed, no vaccine is available against SOSV, however, the fact that 5 years after infection, immune cells isolated from the first infected patient were able to elicit an Ab response is promising for a durable vaccine-induced functional immunity (Parrington et al., 2023). With a robust animal model, several vaccine candidates could be developed such as the mRNA-based NiV vaccine, which is currently in Phase I trial (Mishra et al., 2024).

With several emerging infections on the rise, it is important to investigate whether SOSV could cause a future outbreak, especially given the fact that the ERBs that harbor this virus are known to have a large range: North Africa, Sub-Saharan Africa, the Middle East, Pakistan, and northern India (Orsag et al., 2023). Knowing that ERBs fly up to 32 km and forage on human produce, depositing contaminated feces, urine and saliva, one could speculate that SOSV-harboring bats could be traveling either northward or southward across the African and Asian continents, leading to sporadic outbreaks (Amman et al., 2023). This has been the case of Marburg virus, which has ERBs as its reservoir and has led to 17 sporadic outbreaks until 2023 (Mitu and Islam, 2024). As previously shown, micro-global positioning systems, which install micro-GPS units on ERBs and identify ERB foraging locations, could be used to rapidly monitor and prevent potential outbreaks (Amman et al., 2023), In order to rapidly identify SOSV in affected regions, an inexpensive rapid antigen testing kits, similar to those widely used by the public health agencies in the context of asymptomatic infections during the COVID-19 pandemic, should be developed (Bond et al., 2022; Polechová et al., 2022; Schwartz et al., 2021). Indeed, as of yet, there is no evidence to suggest that SOSV does not cause asymptomatic infections and it would be of interest do determine whether this is also the case for this virus. Moreover, it is also plausible that the situation with the SOSV infections is comparable with the Mayaro virus infection, as its true incidence is likely underestimated and could be mistaken for other acute febrile tropical diseases such as dengue fever (Terzian et al., 2015). Public health professionals must therefore be aware that, due to the lack of awareness regarding SOSV infection, symptoms such as fever, generalized myalgia, arthralgia, headaches, and malaise can be mistakenly diagnosed even in endemic areas. Further, in areas where it may be possible, polymerase chain reaction tests and sequencing of the samples isolated from patients in order to deposit them in a sequence bank, such as the GISAID initiative, which currently tracks a variety of viruses including but not exclusively SARS-CoV-2, Dengue virus (DENV), influenza, etc (Elbe and Buckland-Merrett, 2017).

Determining the exact host cell receptor of SOSV and whether it is conserved across different species would be of great importance, as it would indicate the likelihood of spilling over to humans (Haas and Lee, 2023; Thibault et al., 2017). As SOSV does not appear to bind to sialic acid, it may use a protein or glycoprotein nature receptor that may or may not be similar to other paramyxoviruses like ephrins (henipaviruses), CD150/SLAMF1 and nectin-4 (morbilliviruses), which remains to be seen (Haas and Lee, 2023). Along with the host cell receptor, understanding how SOSV interacts with the host’s immune response could reveal outlying mechanisms of pathogenesis and transmission to other humans (Thibault et al., 2017). It remains to be revealed whether SOSV, an unsegmented virus can cause chronic infections, without being vector-borne, and with a low mortality which are virological factors predicted to increase human-to-human transmissions (Geoghegan et al., 2016). Finally, raising awareness of this emerging zoonotic virus, especially to local populations should be prioritized to minimize their contact with the bats and virus-contaminated fomites, as basic measure to decrease the risks of contamination (Wu et al., 2023).

To conclude, SOSV is a novel zoonotic emerging virus from African ERBs that has caused severe disease in one patient as of yet. The ecology and biology of SOSV should be carefully studied for the purpose of developing the targeted molecular diagnostics and effective antiviral strategies, which currently are lacking. For a future integrative study, an important aspect to consider is the proximity of caves harboring ERBs to densely populated villages (Makenov et al., 2023). Understating how the locals interact with bats whether by their consumption or physical contact, is crucial, since poor hygiene could expose them to SOSV-contaminated saliva, urine or blood (Makenov et al., 2023). The accessibility of healthcare services, diagnosis and prevention will be essential in controlling potential SOSV outbreaks. Education through programs similar to the Tanzania Field Epidemiology and Laboratory Training Program (TFELTP) can be helpful to train healthcare staff in organizing a well-coordinated response in SOSV outbreaks, to trace suspected or confirmed infected cases with daily follow-ups by collaborating with public health officials (Hussein et al., 2024). In this literature review, the progress in SOSV research was highlighted in order to provide further avenues in researching this pararubulavirus, mainly in the development of new animal models, antiviral strategies, and the eventual development of a SOSV vaccine, summarized in Table 1. An integrative One Health approach implemented at both local and international levels could guarantee an effective and timely collective response to this emerging public health threat.

**TABLE 1:** Summary of studies conducted on Sosuga virus (SOSV).

Year	Study conducted	References
2014	First documented case of Sosuga virus (SOSV) in a wildlife biologist.	Albariño et al., 2014
2015	Isolated SOSV from Egyptian rousette bats (ERBs; *Rousettus aegyptiacus*), suggesting this species to be a potential natural reservoir of SOSV.	Amman et al., 2015
2018	Developed a reverse genetics system to allow rapid screening of antiviral compounds.	Welch et al., 2018
2018	Found that antibody-mediated virus neutralization is not a universal mechanism of SOSV in ERBs.	Schuh et al., 2019
2019	Proposed that SOSV may enter host cells using a sialic acid-independent mechanism.	Stelfox and Bowden, 2019
2020	Experimental infection of ERBs with SOSV.	Amman et al., 2020
2021	*In vitro* evaluation of antiviral molecules against several viruses, including SOSV.	Lo Michael et al., 2021
2022	Evaluated histopathology and immunohistochemistry of induced lesions, tissue tropism and host responses in ERBs experimentally infected previously.	Kirejczyk et al., 2022
2023	Initial characterization of experimental infection of SOSV in Syrian hamsters, showing infection, in absence of clinical and histopathological findings in tissues.	Welch et al., 2023
2023	Developed potently neutralizing human monoclonal antibodies (mAbs) against SOSV.	Parrington et al., 2023
2024	Detection of SOSV RNA in ERBs captured from Sierra Leone.	Amman et al., 2024
